# Mediastinal undifferentiated pleomorphic sarcoma with pleural effusion cytopathologically misdiagnosed as epithelial malignant pleural mesothelioma: An autopsy case report

**DOI:** 10.1111/1759-7714.13898

**Published:** 2021-02-19

**Authors:** Kinnosuke Matsumoto, Yukihiro Nakamura, Yuji Inagaki, Yoshihiko Taniguchi, Akihiro Tamiya, Yoshinobu Matsuda, Takahiko Kasai, Shinji Atagi

**Affiliations:** ^1^ Department of Internal Medicine National Hospital Organization Kinki‐Chuo Chest Medical Center Osaka Japan; ^2^ Department of Pathology National Hospital Organization Kinki‐Chuo Chest Medical Center Osaka Japan; ^3^ Clinical Research Center National Hospital Organization Kinki‐Chuo Chest Medical Center Osaka Japan

**Keywords:** cell block, malignant pleural mesothelioma, mediastinum, pleural effusion, undifferentiated pleomorphic sarcoma

## Abstract

Undifferentiated pleomorphic sarcoma (UPS) is a new disease in the World Health Organization's classification of tumors of soft tissue and bone published in 2013. Primary mediastinal UPS is rare, especially with pleural effusion. Herein, we describe the pathological findings of pleural effusion followed by mediastinal UPS, which was initially misdiagnosed as epithelial malignant pleural mesothelioma (MPM). The cytopathological findings of the pleural effusion cell block often contribute to the diagnosis of various malignant tumors. However, these findings may lead to misdiagnosis of highly invasive mediastinal tumors such as UPS. A biopsy for primary mediastinal lesions should be performed because MPM rarely mimics mediastinal tumors with pleural effusion.

## Graphical abstract

Mediastinal undifferentiated pleomorphic sarcoma (UPS) is rare, especially with pleural effusion. Herein, we describe the pathological findings of pleural effusion followed by mediastinal UPS, cytopathologically mimicking epithelial malignant pleural mesothelioma.

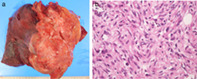

## INTRODUCTION

Undifferentiated pleomorphic sarcoma (UPS), previously known as malignant fibrous histiocytoma (MFH), was reclassified into an unclassifiable/undifferentiated sarcoma (US) in the fourth edition of the World Health Organization's classifications published in 2013.^1^ UPS is common in malignant soft tissue tumors and occurs in the extremities and retroperitoneum.^2^ Primary mediastinal UPS is rare, especially with pleural effusion. Therefore, the characteristics of pleural effusion with UPS remain unclear.^3^ We report a case of mediastinal UPS with pleural effusion cytopathologically misdiagnosed as epithelial malignant pleural mesothelioma (MPM).

## CASE REPORT

**FIGURE 1:**
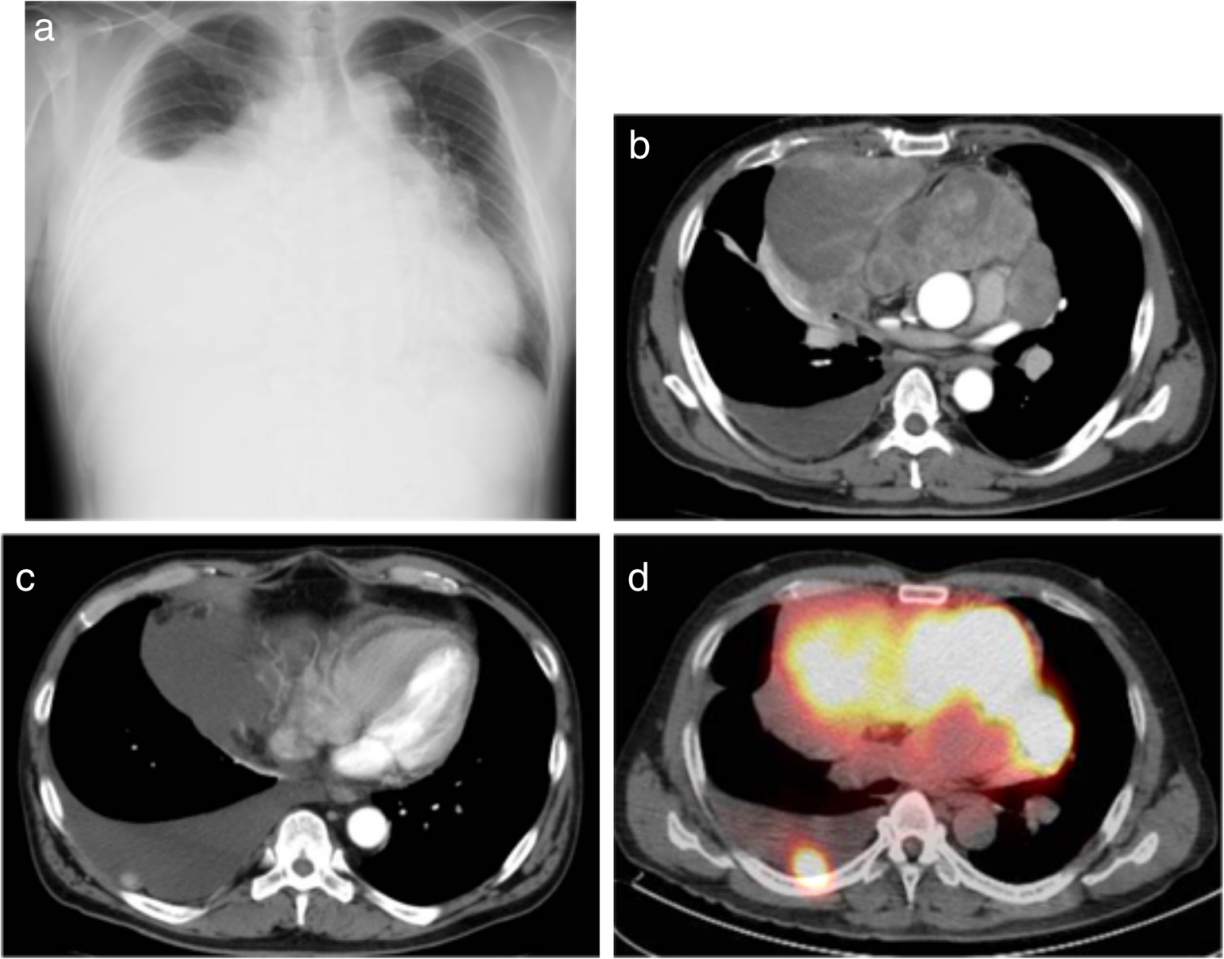
Images from the previous hospital. (a) Chest X‐ray showed an extensive abnormal shadow in the right lower lung field and cardiac expansion. (b, c) Chest computed tomography (CT) revealed moderate right pleural effusion, nodules in the pleura, and a huge tumor at the anterior mediastinum. (d) Positron emission tomography CT detected abnormal high uptake of fluorodeoxyglucose in the mediastinal tumor and nodules in the pleura

**FIGURE 2:**
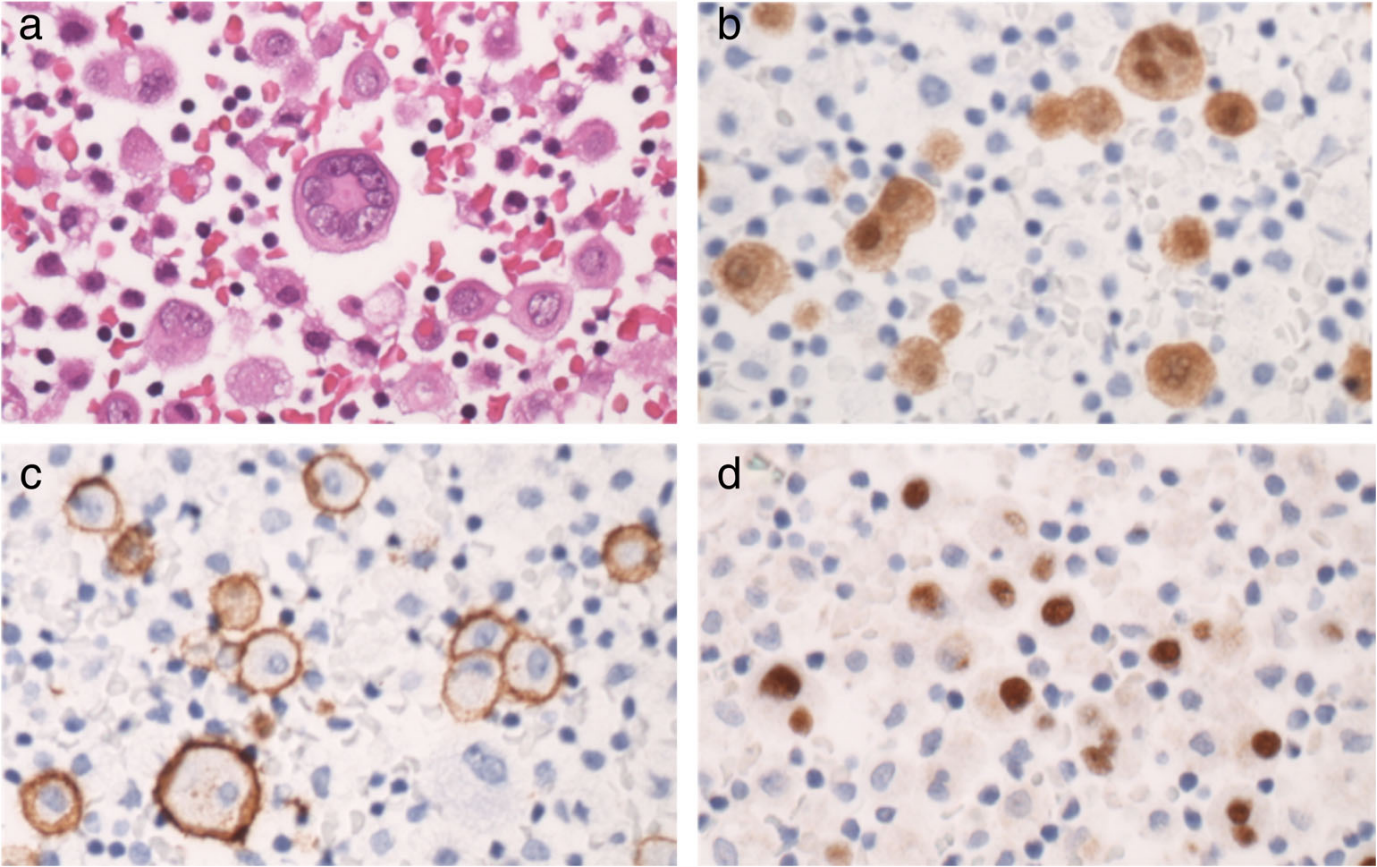
Hematoxylin and eosin (HE) and immunohistochemistry (IHC) staining of cell block sample. (a) HE staining showed some multinucleated and atypical mesothelial cells with hump‐like cytoplasmic processes and window formation (40 × 10). (b–d) IHC staining showed positive calretinin, D2‐40, and WT‐1 (40 × 10)

A 50‐year‐old man with acute onset of dyspnea visited another hospital. He had an unremarkable medical history. However, he had smoked two packets of cigarettes per day for 25 years and had been in the construction industry with significant asbestos exposure since he was 20 years old. Chest radiography showed extensive abnormal shadows in the right lower lung field and cardiac expansion (Figure 1a). Chest computed tomography (CT) revealed moderate right pleural effusion, two pleural nodules, and a large mediastinal tumor invading the pericardium (Figure 1b,c). Aside from the mediastinal tumor and pleural nodules, no further abnormal fluorodeoxyglucose uptakes were detected by positron emission tomography CT (Figure 1d). The pleural effusion was drained. The cell block sample showed some multinucleated and atypical mesothelial cells with hump‐like cytoplasmic processes among lymphocyte inflammation upon hematoxylin–eosin (HE) staining (Figure 2a). On immunohistochemistry (IHC) staining, calretinin, D2‐40, WT‐1, and HEG1 were positive (Figure 2b–d) while TTF‐1, CEA, claudin‐4, and desmin were negative. Based on these findings, MPM was suspected. The patient was then referred to our hospital.

The patient was urgently hospitalized because of severe tachycardia and hypoxia at his initial visit. Laboratory findings, including tumor markers, such as CEA, CYFRA, and ProGRP, were almost within normal limits, except for high levels of D‐dimer (1.2 × 10^4^ ng/ml) and CRP (0.15 g/l). Transthoracic echocardiography showed that both ventricles were pushed directly by the tumor and had almost collapsed.

**FIGURE 3:**
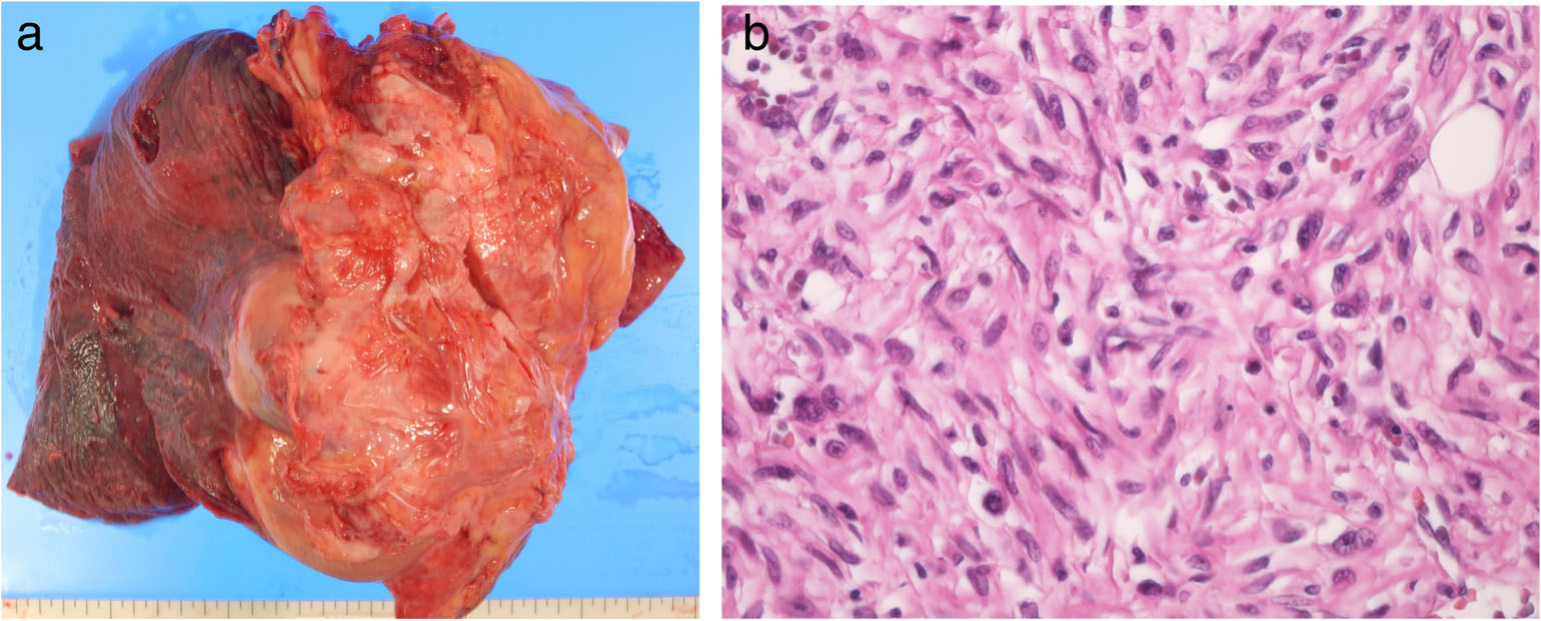
(a) Autopsy revealed that the specimen resected from the mediastinum was 160 × 150 × 140 mm in size and invaded the pericardium. (b) Hematoxylin and eosin staining of the mediastinal specimen showed atypical fibroblast‐like cells were arranged in a storiform pattern (40 × 10)

A diagnostic percutaneous needle biopsy was performed. Polymorphic variant cells with nonspecific differentiation were observed and almost all markers, including calretinin, D2‐40, and WT‐1, were negative. The histological findings of the mediastinal tumor were completely different from those of pleural effusion. This did not lead to a definitive diagnosis of MPM. After a few days, the patient died of untreated cardiac tamponade. An autopsy revealed that atypical fibroblast‐like cells were arranged in a storiform pattern. This was associated with a pleomorphic feature (Figure 3a,b). Immunohistochemically, tumor cells were negative for almost all markers such as CK‐AE1/AE3, CK‐CAM5.2, EMA, calretinin, desmin, α‐SMA, D2‐40, CD34, S‐100, and neurofilament. This finding was similar to the needle biopsy sample. Thus, we diagnosed the patient with UPS.

## DISCUSSION

MFH was first described in 1963 as a malignant soft tissue tumor arising from histiocytes and characterized by a storiform pattern with less differentiated and pleomorphic formation.^4^ MFH was regarded as the most common soft tissue sarcoma in adults for many years. However, Fletcher and Daugaard reported that most tumors diagnosed as MFH turned out to be other types of sarcomas upon using techniques such as immunochemistry.^5^, ^6^, ^7^ Therefore, the term MFH was completely removed from the 2013 WHO Classification and replaced with a new category, US, which pertained to tumors that did not show a specific differentiation pattern despite various examinations. Moreover, US was classified into five subtypes. The UPS subtype displays polymorphism.

Since the revision of the WHO classification in 2013, only four cases of primary mediastinal UPS have been reported. All cases had undifferentiated and pleomorphic patterns on HE and IHC staining.^3^, ^8^, ^9^, ^10^ Generally, UPS is an exclusion diagnosis. Our case was consistent with the aforementioned findings. Although one of the four cases had pleural effusion, pathological evaluation was not performed. Mehta and Guardiola reported a case of pleural MFH with pleural effusion, and the CT findings resembled MPM closely. However, the cell block of pleural effusion was not evaluated.^11^

**FIGURE 4:**
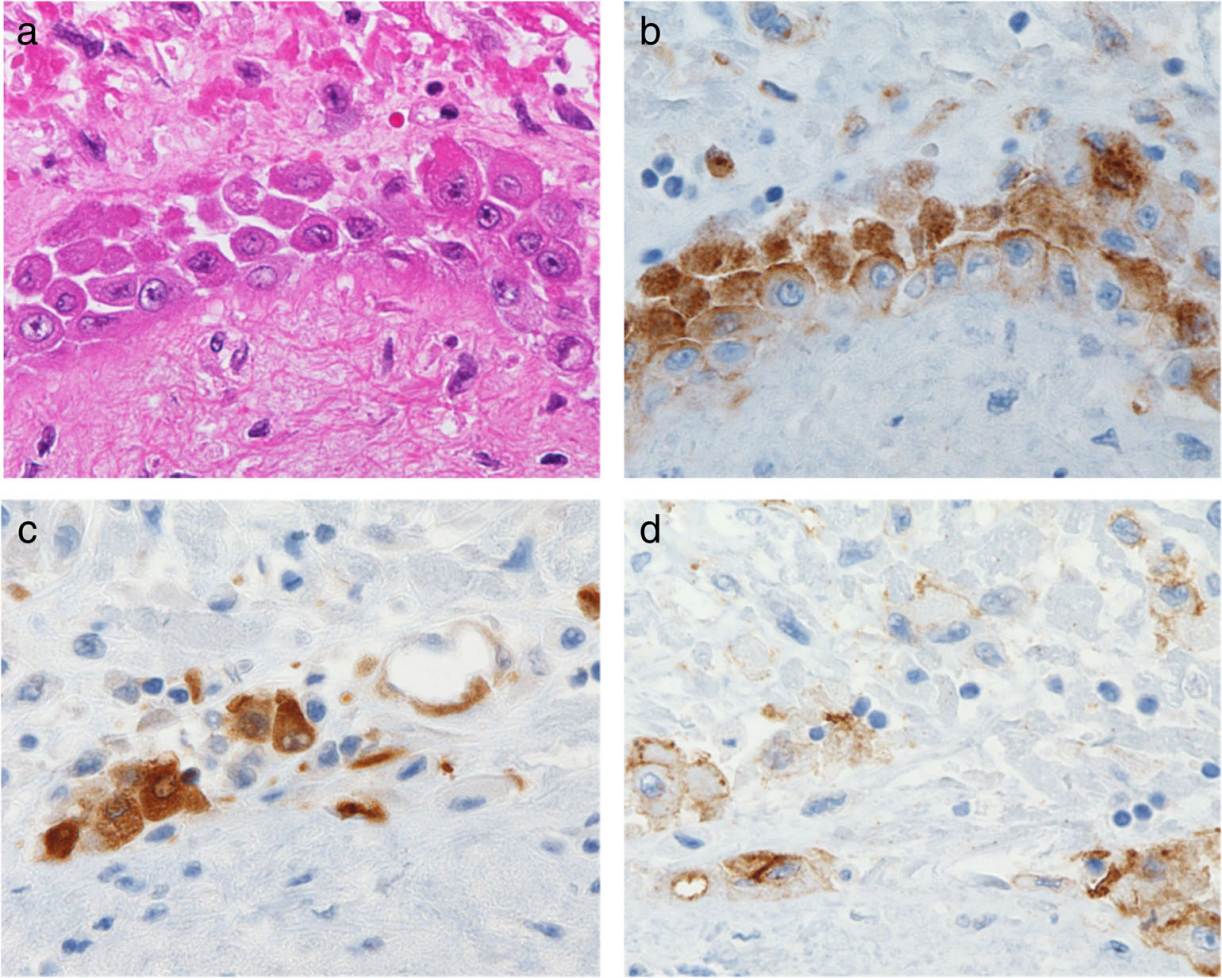
Hematoxylin and eosin (HE) and immunohistochemistry (IHC) staining of mediastinal sample. (a) HE staining showed atypical mesothelial cells in the pericardium (40 × 10). (b–d) IHC staining showed HEG1, calretinin, and D2‐40 were positive for atypical mesothelial cells (40 × 10)

Therefore, we discussed the histological findings of pleural effusion associated with mediastinal UPS. In this case, although MPM was not generally suspected from the radiological findings, both MTAP and BAP1 on IHC were positive and homozygous deletion of 9p21 on fluorescence in situ hybridization was difficult to determine in the cell block sample; despite being rare, mediastinal MPM was also identified.^12^, ^13^, ^14^ The autopsy revealed few asbestos bodies, and the tumor directly invaded the mesothelium of the pericardium and pleura, producing reactive mesothelial cells (Figure 4a–d). Therefore, the reason for differences between the findings of the cell block sample and the mediastinal tumor was revealed, and MPM was almost ruled out.

The general prognosis for mediastinal UPS is poor because of its rapid progression and the difficulty of complete resection of the primary lesion in the mediastinum.^15^ Therefore, early diagnosis and treatment are required. Thoracentesis is easier to perform than biopsy of the primary mediastinal lesion, especially for a patient with severe cardiopulmonary status such as the current case. The pathological findings of the pleural effusion cell block often contribute to the diagnosis of various malignant tumors. However, these findings may lead to misdiagnosis of highly invasive mediastinal tumors such as UPS. Moreover, MPM rarely mimics mediastinal tumors with pleural effusion and a biopsy of the primary mediastinal lesion should be performed.

## CONFLICT OF INTEREST

Dr Tamiya reported grants and personal fees from AstraZeneca, Ono Pharmaceutical, and Bristol‐Myers Squibb; personal fees from Eli Lilly Japan, Chugai Pharmaceutical, Boehringer Ingelheim, MSD, Pfizer, Taiho Pharmaceutical, and Kissei Pharmaceutical. Dr Atagi reported grants and personal fees from AstraZeneca, Ono Pharmaceutical, Bristol‐Myers Squibb, Eli Lilly Japan, Boehringer Ingelheim, MSD, Pfizer, Taiho Pharmaceutical, Chugai Pharmaceutical, and Merck Pharmaceutical; grants from F. Hoffmann‐La Roche; personal fees from Kyowa Hakko Kirin, and Hisamitsu Pharmaceutical. The remaining authors state that they have no conflict of interest.
